# Brain magnetic resonance spectroscopy and cognitive impairment in chronic hepatitis C patients

**DOI:** 10.1186/s41983-018-0046-7

**Published:** 2018-12-20

**Authors:** Ahmed Abo Hagar, Youssri Ashour, Mohamed Negm, Mohamed Abdelfatah, Khaled A. Gad, Ehab Hashish

**Affiliations:** Faculty of Medicine, Suez Canal University, Ismailia, Egypt

**Keywords:** Chronic hepatitis C, Hepatic encephalopathy, MRS, Cognitive dysfunction

## Abstract

**Background:**

Cognitive dysfunction in patients with chronic hepatitis C virus (HCV) infection may appear long before the development of severe liver cirrhosis. These alterations are not ascribed to hepatic encephalopathy; however, early detection is always difficult.

**Objective:**

The aim of this study was to assess the changes of magnetic resonance spectroscopy (MRS) metabolites among chronic hepatitis C virus patients with and without cognitive impairment.

**Patients and methods:**

A cross-sectional study was conducted in Suez Canal University Hospital. Forty-six HCV patients was included and divided into two groups: patients with and without cognitive impairment. Assessment of cognitive function was done using mini-mental state examination and Wechsler Memory Scale - Revised. Both groups were subjected to single-voxel MRS to evaluate metabolites in three brain regions: the basal ganglia, hippocampus, and posterior cingulate gyrus.

**Results:**

The CHO/Cr was significantly higher, and NAA/Cr was significantly lower in group with cognitive impairment in the basal ganglia and posterior cingulate gyrus. Mini-mental state score had negative significant correlation with PCR of HCV. Mini-mental state score had significant negative and positive correlation with CHO/Cr and NAA/Cr, respectively, in the basal ganglia. All values of the Wechsler Memory Scale were statistically higher in the group without cognitive impairment except verbal memory score.

**Conclusion:**

There were changes at brain metabolites associated with cognitive impairment in chronic hepatitis C patients regarding a decrease of NAA/Cr ratio and an increase of CHO/Cr ratio at the basal ganglia.

## Introduction

Hepatitis C virus (HCV) infection is common in Egypt. The prevalence of antibody to HCV in the general population is around 15–20% [1]. Alterations in cerebral functions in chronic HCV infection patients may appear long before the development of severe liver cirrhosis in the absence of hepatic encephalopathy. Irrespective of the severity of liver disease, about 50% of patients with chronic HCV infection showed neuropsychiatric symptoms leading to some degree of quality of life impairment [2]. Hilsabeck et al. (2003) [3] have documented neurologic abnormalities and cognitive impairments among HCV positive individuals without advanced liver disease. The HCV is thought to cross the blood brain barrier primarily by infecting surrounding monocytes and progenitor cells [4].

Studies using proton H magnetic resonance spectroscopy (H-MRS) showed decrease levels of *N*-acetyl aspartate (NAA) in frontal white matter and increase in myo-inosital (mI) and choline/creatine values in the basal ganglia in chronic hepatitis C patients. It is not known if these changes are related to cognitive impairment in chronic hepatitis C patients [5]. Yet, the information on the biochemical and functional changes of the brain in those patients is limited [6].

The aim of this study was assessing of changes of MRS metabolites among chronic hepatitis C virus patients with and without cognitive impairment.

## Subjects and methods

A cross-sectional study was conducted in Suez Canal university hospital. The study included 46 patients with compensated chronic hepatitis C-positive patients who were eligible to antiviral therapy but not starting with, with high education level (final stage after completion of secondary education), age range 18–65 years, and both genders were included. We excluded patients with renal failure, decompensated liver disease, hypoxic encephalopathy, drug or alcohol intoxication, history of old cerebrovascular stroke, seizures, patient with pacemaker, or metal implant.

Patients were divided into two groups: patients with HCV with and without cognitive impairment. Both groups were matched for age, sex, and staging.

Included subjects were subjected to complete medical history and full abdominal and neurological examination. Laboratory investigations were done including liver function tests, renal function, and PCR for HCV.

Brain MRI and MRS was done for each subject, a high-resolution sagittal T1-weighted MR scan was acquired by using a 1.5 T Philips Achieva scanner, TR = 20 ms, TE = 5 ms, flip angle = 30, field of view = 220 mm, acquisition matrix 256, and slice thickness 1.3 mm. All subjects were investigated using cerebral MRI and MRS studies using single-voxel technique of short-time echo to identify metabolites of interest. The spectroscopic voxel of interest was positioned to assay metabolites in three brain regions: the basal ganglia (putamen and globuspallidus combined), posterior cingulate gyrus, and hippocampus. Metabolites of interest were choline (CHO), creatine (Cr), glutamine-glutamate (Glu), myo-inisotol (MYO), and *N*-acetyl aspartate (NAA). This attempt was to determine mainly the choline/creatine ratios (CHO/Cr ratio), myo-inistol to creatine ratio (MYO/Cr), *N*-acetyl aspartate-to-choline ratio (NAA/CHO ratio), glutamate to creatine ratio (Glu/Cr ratio), and *N*-acetyl aspartate-to-creatine ratios (NAA/Cr ratio).

### Assessment of cognitive functions

It was assessed by mini-mental state examination as screening tool for categorization of patients before imaging and Wechsler Memory Scale - Revised, the short form for further details after imaging.

### Mini-mental state examination (MMSE) [7]

The mini-mental state examination is probably the most widely used measure of cognitive decline. The MMSE has a maximum score of 30 points; patients with MMSE > 28 were recruited in chronic hepatitis C patients without cognitive impairment group, while patients with MMSE scores 25–28 [8] were recruited in chronic hepatitis C patients with cognitive impairment group, patients with MMSE of 24 or less were excluded.

### Wechsler Memory Scale - Revised (WMS-R) short form [9]

The Wechsler Memory Scale - Revised short form comprises a series of brief subtests, each measuring a different facet of memory functions. Accordingly, WMS-R results are thought to measure the cognitive performance as follows: 1-verbal memory, 2-visual memory, 3-attention/concentration/psychomotor speed assessment, and 4-visuo-spatial function assessment. The test takes around 45 min. The subtests that were presented to the subjects as verbal stimuli were available as an Arabic version.

### Statistical analysis

Data was managed using Statistical Package of Social Sciences (SPSS) version 16.0. Statistical significance tests were used and *p* value of less than or equal (0.05) was considered statistically significant (at 95% level of confidence). Descriptive statistics were presented as (mean ± standard deviation) for quantitative variables and as (%) for qualitative variables. Correlation was done using Pearson coefficient to assess relationships between quantitative variables. ANOVA test was used to compare means of metabolite ratios among different brain regions.

### Ethical consideration

An informed written consent was taken from each patient. All data obtained from every patient were confidential and were not used outside the study. The patients have the rights to withdraw from the study at any time without giving any reason. All the cost of the investigations was afforded by the researcher.

The study was approved from ethical committee Faculty of Medicine, Suez Canal University on January 14, 2015 (research no. 2450).

## Results

This study included 46 patients with chronic hepatitis c virus, 23 patients with cognitive impairment, and 23 patients without cognitive impairment. The mean age was 53.8 ± 7.4 and 47.7 ± 13.8 for the group with and without cognitive impairment, respectively. In group without cognitive impairment, 47.8% were males and 52.2% were females, while in group with cognitive impairment, 43.5% were males and 56.5% were females (Table 1). The mean of MMSE in group without cognitive impairment was 30.0 **±** 0.0, and for group with cognitive impairment was 26.6 **±** 0.89 (Table 1). The mean PCR for group without cognitive impairment was (45,391.3 **±** 44,016.2), and for group with cognitive impairment was (685,217.3 **±** 222,176.6) (Table 1).

**Table 1:** Demographic data in both groups

		Group without cognitive impairment *N* = 23		Group with cognitive impairment *N* = 23		*p* value
Age	Mean ± SD	47.7 ± 13.8		53.8 ± 7.4		0.068 ^a^
Gender	Male	11	47.8%	10	43.5%	0.500 ^b^
	Female	12	52.2%	13	56.5%	
MMSE	Median	30		27		0.000
	IQ range			1		
	Mean ± SD	30.0 ± 0.0		26.6 ± 0.89		
	Range	30		25–28		
PCR	Mean ± SD	45,391.3 ± 44,016.2		685,217.3 ± 222,176.6		0.000 ª

*NS* no statistically significant difference

^a^Student *t* test

^b^Chi-square test

In the basal ganglia, the CHO/Cr and the NAA/Cr were significantly higher in the group with cognitive impairment (Table 2).

**Table 2:** Basal ganglia metabolites ratios in both groups

		Group without cognitive impairment Mean ± SD (*N* = 23)	Group with cognitive impairment Mean ± SD (*N* = 23)	*p* value
BG	Glu/Cr	0.45 ± 0.09	0.46 ± 0.10	0.784 NS^α^
	CHO/Cr	0.89 ± 0.14	1.00 ± 0.10	0.000 *^α^
	MI/Cr	0.51 ± 0.10	0.55 ± 0.09	0.122 NS^α^
	NAA/CHO	1.86 ± 0.36	1.83 ± 0.30	0.854 NS^α^
	NAA/Cr	1.59 ± 0.39	1.39 ± 0.31	0.011 *^α^

*NS* non-statistically significant difference, *BG* basal ganglia

^α^Mann-Whitney test

*Statistically significant difference

In the hippocampus, no significant differences existed between both groups regarding metabolites despite that NAA/Cr was higher in patients without cognitive impairment.

**Fig. 1:**
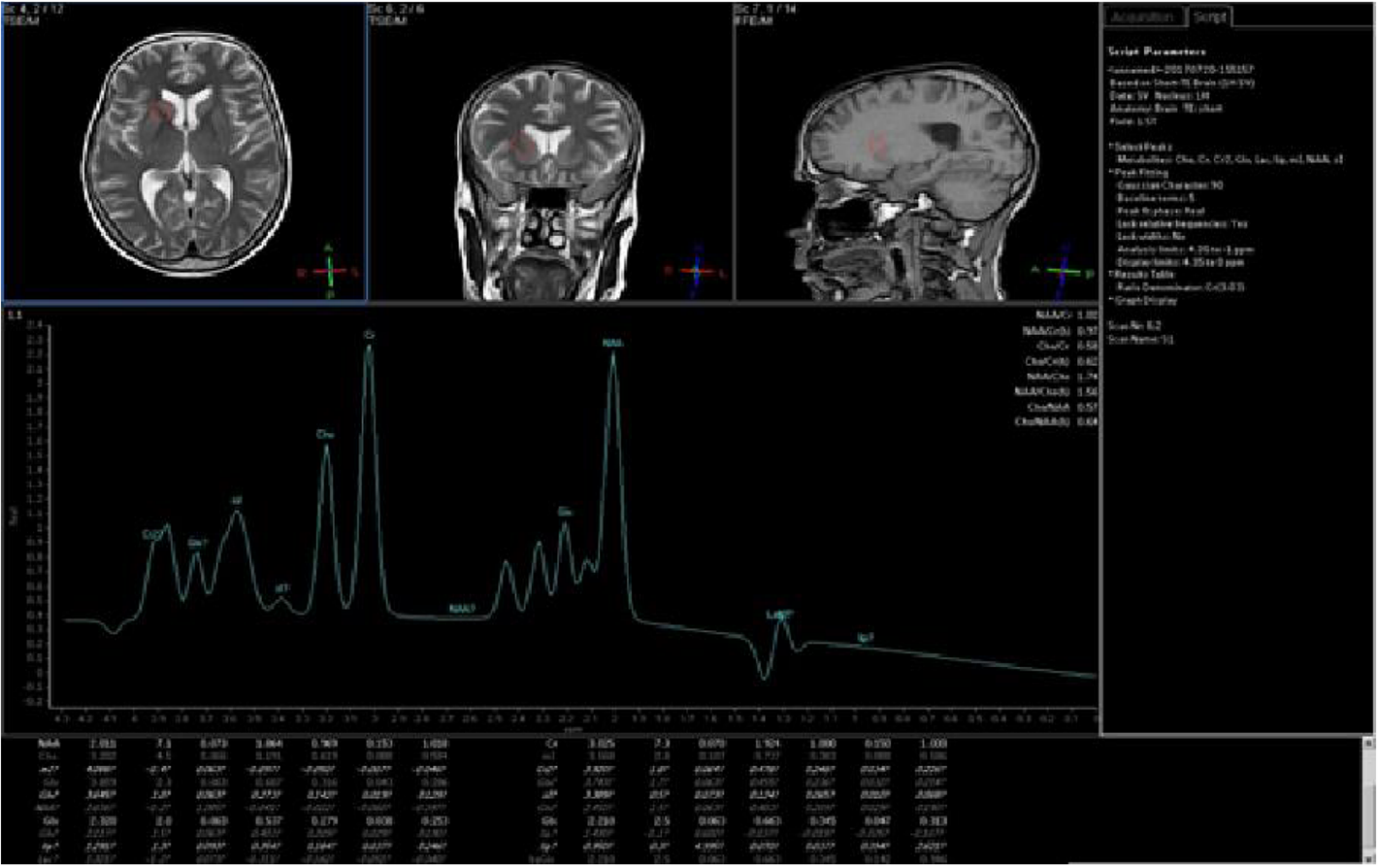
MRS at basal ganglia in patients with cognitive impairment. Spectroscopic voxel of interest were done at basal ganglia in sagittal, coronal, and axial plans in patients with impaired attention/concentration/psychomotor score. Curve showed that NAA/Cr ratio was elevated (*R* = 0.499 and *p* value = 0.026), and CHO/Cr ratio was reduced (*R* = − 0.630 and *p* value = 0.008)

**Fig. 2:**
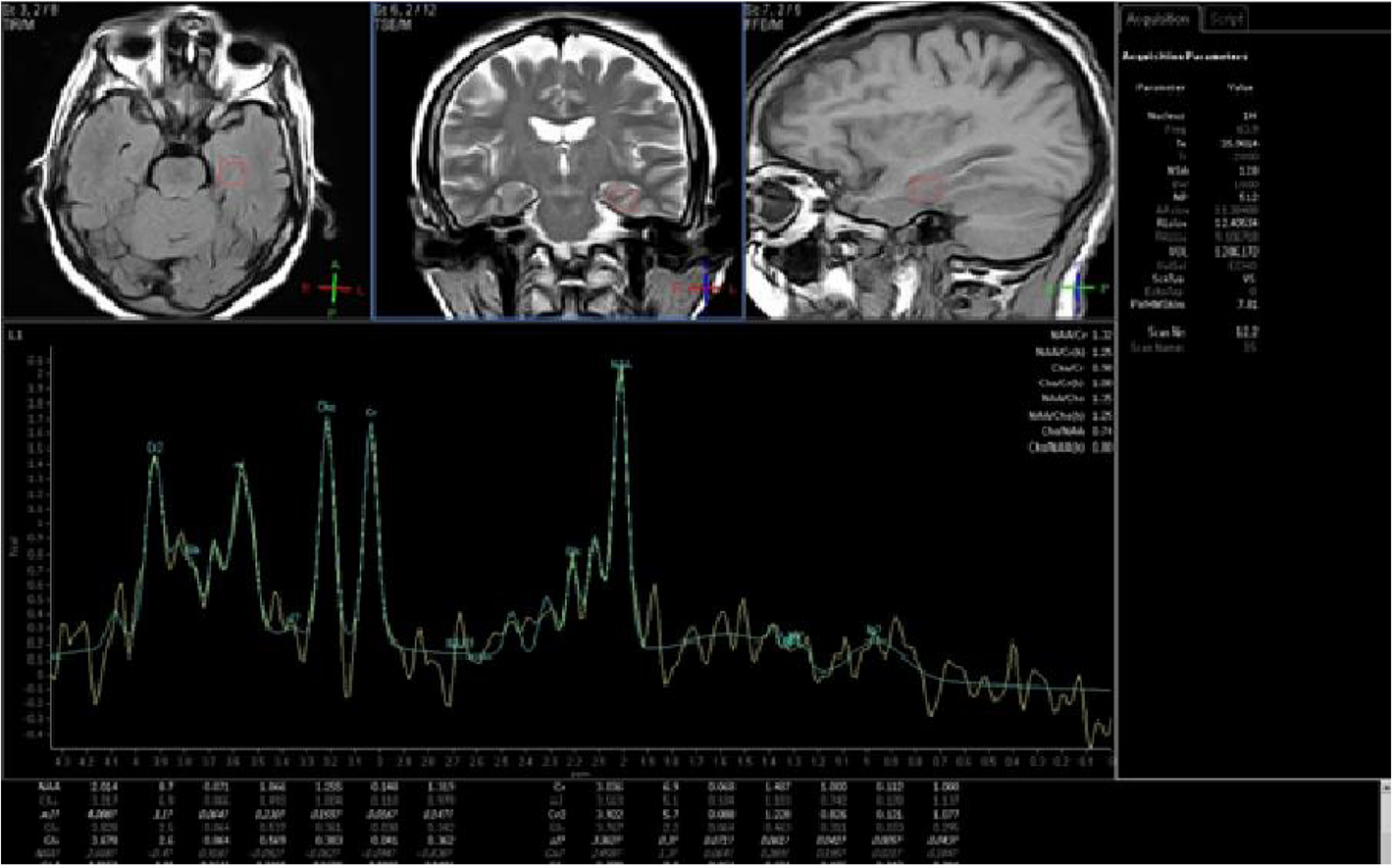
MRS at the hippocampus in patients with cognitive impairment. Spectroscopic voxel of interest were done at the hippocampus in sagittal, coronal, and axial plans in patients with impaired visual memory score. Curve showed that CHO/Cr ratio was reduced (*R* = − 0.445 and *p* value = 0.033)

**Fig. 3:**
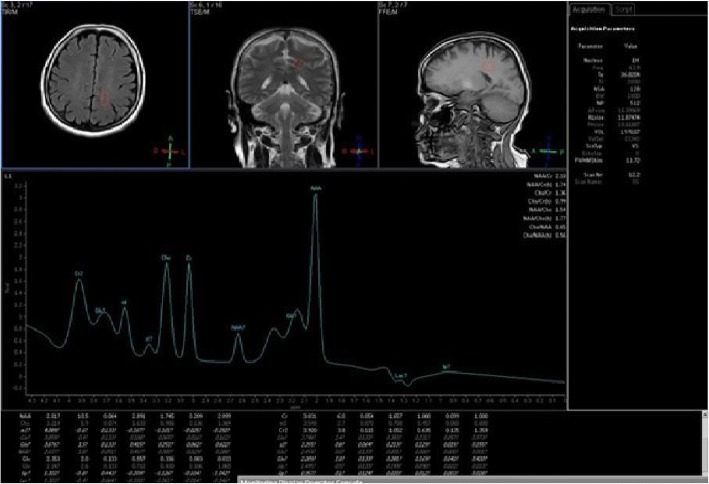
MRS at posterior cingulate gyrus in patients with cognitive impairment. Spectroscopic voxel of interest was done at posterior cingulate gyrus in sagittal, coronal, and axial plans in patients with impaired Visuo-spatial function score. Curve showed that NAA/Cr ratio was elevated (*R* = 0.409 and *p* value = 0.009)

In posterior cingulate gyrus, the CHO/Cr ratio was significantly higher and NAA/Cr was significantly lower in the group with cognitive impairment (Table 3) (Figs. 1, 2, and 3).

**Table 3:** Posterior cingulate gyrus metabolite ratios in both groups

		Group without cognitive impairment Mean ± SD (*N* = 23)	Group with cognitive impairment Mean ± SD (*N* = 23)	*p* value
Cingulate gyrus	Glu/Cr	0.44 ± 0.17	0.43 ± 0.16	0.913 NS^α^
	CHO/Cr	0.86 ± 0.17	0.91 ± 0.16	0.019*^α^
	MI/Cr	0.57 ± 0.21	0.57 ± 0.11	0.167 NS^α^
	NAA/CHO	1.95 ± 0.27	1.91 ± 0.22	0.582 NS^α^
	NAA/Cr	1.62 ± 0.30	1.51 ± 0.19	0.021* ^a^

*NS* non-statistically significant difference

^α^Mann-Whitney test

^a^Student *t* test

*Statistically significant difference

**Fig. 4:**
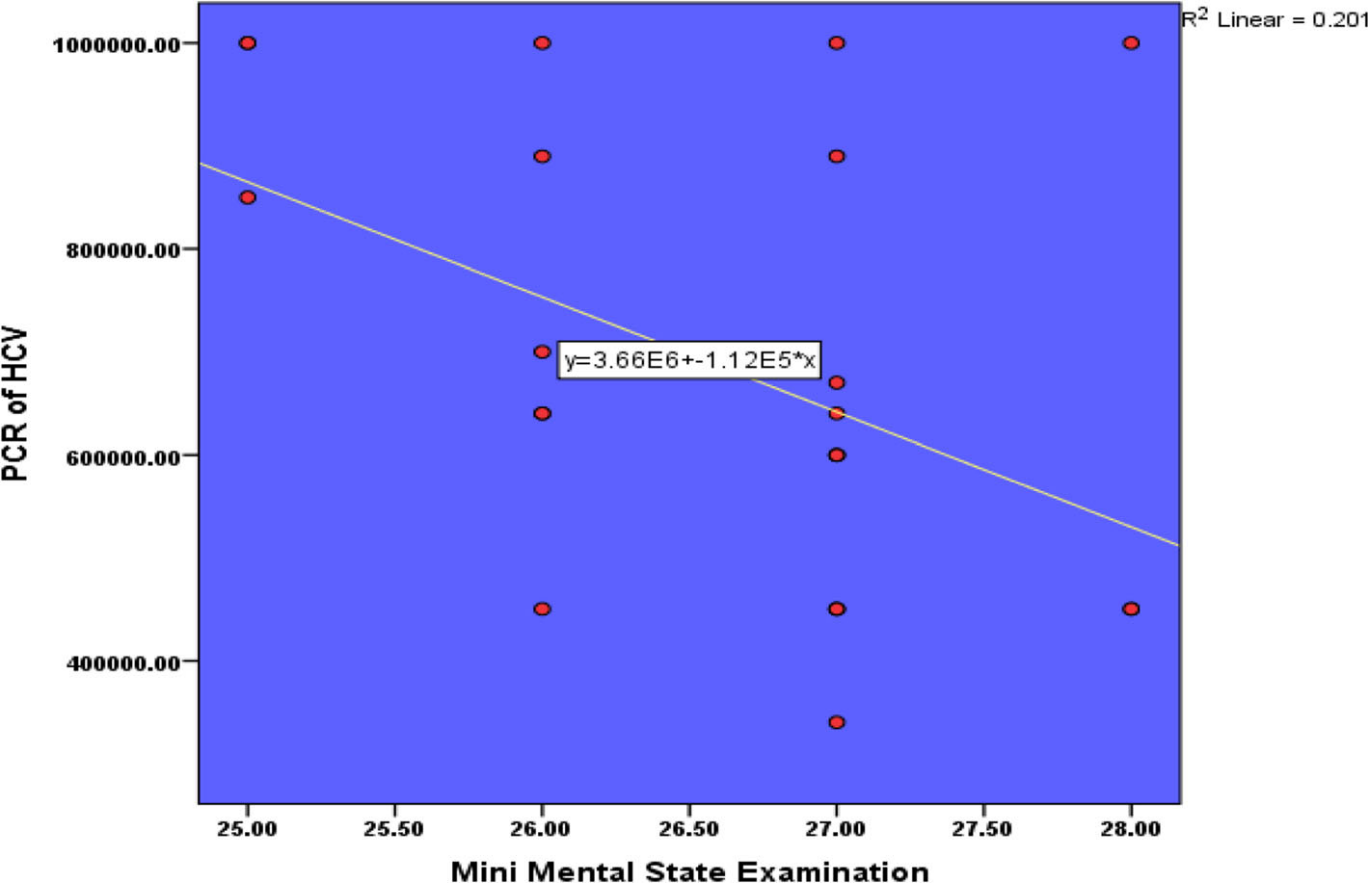
Mini-mental state score and PCR of HCV in the study group

Mini-mental state score had negative significant correlation with PCR of HCV; however, increase virus load was associated with decrease in MMSE score (*p* value = 0.032) (Table 4) and (Fig. 4).

**Table 4:** Mini-mental state score and PCR of HCV in the study group

	Mini-mental state score	
	*r*	*p* value
PCR HCV	− 0.448	0.032 *

*Statistically significant difference-Pearson correlation

Mini-mental state score had significant negative and positive correlation with CHO/Cr and NAA/Cr, respectively, in the basal ganglia (Table 5). On the other hand, mini-mental state score had no significant correlation with any metabolites of the hippocampus or posterior cingulate gyrus.

**Table 5:** Mini-mental state score and basal ganglia metabolites in the study group

		Mini-mental state score	
		*r*	*p* value
BG	Glu/Cr	0.156	0.478 NS
	CHO/Cr	− 0.423	0.013*
	MI/Cr	0.048	0.827 NS
	NAA/CHO	− 0.083	0.707 NS
	NAA/Cr	0.610	0.029*

*NS* non-statistically significant difference

*Statistically significant Pearson correlation

All values of the Wechsler Memory Scale components were statistically higher in the group without cognitive impairment compared to group with cognitive impairment except verbal memory score (Table 6).

**Table 6:** Wechsler Memory Scale in both groups

	Group without cognitive impairment *N* = 23	Group with cognitive impairment *N* = 23	*p* value
Visual memory assessment	66.47 ± 2.01	60.82 ± 2.16	0.000 *^α^
Verbal memory assessment	51.91 ± 1.34	48.04 ± 1.79	0.253 NS^a^
Attention/concentration/psychomotor	27.95 ± 1.36	21.73 ± 1.88	0.000 *^α^
Visuo-spatial function assessment	40.08 ± 0.90	34.0 ± 2.43	0.000 ^α^*

^α^Mann-Whitney test

^a^Student *t* test

*Statistically significant difference

Verbal memory score had no significant correlation with any metabolites of the basal ganglia or hippocampus. Verbal memory assessment score had only negative significant correlation with CHO/Cr ratio in cingulate gyrus (*R* = − 0.424 and *p* value = 0.044), no significant correlation with any metabolites of the basal ganglia or hippocampus.

Visual memory score had no significant correlation with any metabolites of the basal ganglia. On other hand, visual memory assessment score had negative significant correlation with CHO/Cr (*R* = − 0.445 and *p* value = 0.033) and Mi/Cr (*R* = − 0.513 and *p* value = 0.012) ratio in the hippocampus. Also, visual memory assessment score had significant positive correlation with NAA/Cr (*R* = 0.553 and *p* value = 0.024) in posterior cingulate gyrus.

In the basal ganglia of the study group, attention/concentration/psychomotor score had significant negative and positive correlation with CHO/Cr (*R* = − 0.630 and *p* value = 0.008) and NAA/Cr (*R* = 0.499 and *p* value = 0.026), respectively. On the other hand, attention/concentration/psychomotor score had no significant correlation with any metabolites of the hippocampus or cingulate gyrus.

Visuo-spatial function assessment score had a significant positive correlation with NAA/Cr (*R* = 0.409 and *p* value = 0.009) in both cingulate gyrus areas. It had no significant correlation with all metabolites in basal ganglia and hippocampus (Figs. 1, 2, and 3).

## Discussion

In this study, we compared between brain metabolites using single-voxel brain magnetic resonance spectroscopy in chronic hepatitis C virus patients with and without cognitive impairment. We found that the Cho/Cr ratio was elevated significantly in the basal ganglia and posterior cingulate gyrus in group with cognitive impairment. In contrast, there was a significant decrease of NAA/Cr ratio in those patients. At hippocampal regions, there were no significant differences between both groups. Elevations in cerebral CHO and reduction in NAA in white matter suggested decreasing neuronal activity and glial inflammation and activation [10]. On the contrary from hepatic encephalopathy where the CHO/Cr ratio is reduced, suggesting a different mechanism underlies the findings in HCV infection [11].

Our findings were in agreement with Weissenborn and his colleagues [6]. They found that NAA/Cr signal ratio is reduced over the occipital gray matter. The change in NAA/Cr is more likely to be multifactorial due to viral load and cognitive impairment [6]. Other studies also demonstrated elevations in cerebral CHO and reduction in NAA/Cr in white matter and basal ganglia [10, 12–14].

On the other hand, Forton and his colleagues in 2008 [15] and Thames and colleagues in 2015 [16] found an elevation of mI/Cr ratio in cerebral white matter in chronic hepatitis C patients. They demonstrated a positive correlation between frontal white matter mI and overall cognitive performance. Also, they found elevation of NAA in the basal ganglia. In our study, we did not find statistically significant difference regarding mI/Cr ratio between both groups. This difference may be a result of MRS parameter differences and different methods between studies.

There was a significant negative correlation regarding HCV load by PCR and mini-mental state examination. This finding is in agreement with previous studies which proved that HCV infects the brain cells and may be associated with a neuro-inflammatory process that leads to impairment of cognitive function in compensated hepatitis C virus patients without history of hepatic encephalopathy [17–19]. The mechanism of cognitive impairment is related to change at the level of brain neurotransmitters due to neuro-inflammation and immune response [16].

In studying the correlation between mini-mental state examination and brain metabolites in the group with cognitive impairment, we found significant positive correlation with NAA/Cr and significant negative correlation with CHO/Cr ratio at the basal ganglia study site while there were no significant correlations at the hippocampus and posterior cingulate gyrus study sites. These results agreed with previous studies [15, 16, 20].

These findings were associated with poor cognitive function regarding working memory, psychomotor speed, and reaction time. These findings were explained by microglial activation due to viral infection leading to neuro-inflammation and changes at the level of brain neurotransmitters [21].

Regarding Wechsler Memory Scale and its correlation with brain metabolites, we found a significant positive correlation of visual memory subtests with NAA/Cr at the posterior cingulate gyrus, negative significance with CHO/Cr and Mi/Cr at the hippocampus in the group with cognitive impairment. Furthermore, verbal memory showed negative significance with CHO/Cr ratio at posterior cingulate study site. Attention, concentration, and psychomotor speed subtests of Wechsler Memory Scale showed a significant positive correlation with NAA/Cr and significant negative correlation with CHO/Cr ratio at the basal ganglia study site while hippocampus and posterior cingulate gyrus study sites were statistically insignificant. Visuo-spatial function subtest showed a significant positive correlation with NAA/Cr at posterior cingulate gyrus and non-significant correlation with basal ganglia and hippocampus study sites.

These findings were in agreement with Martindale et al. [21] as NAA/Cr was lower in chronic hepatitis C patients with cognitive impairment at frontal white matter and basal ganglia with elevation of CHO/Cr at the basal ganglia study site.

In conclusion, altered cognition and neuropsychological performance are frequently found in chronic hepatitis C patients without hepatic encephalopathy. These changes typically occur without structural brain abnormality by magnetic resonance imaging of the brain in those patients. There is a significant negative correlation with PCR virus load of HCV and cognitive function. There are changes at brain metabolites associated with cognitive impairment in chronic hepatitis C patients regarding decrease NAA/Cr ratio, increase of CHO/Cr ratio at the basal ganglia. Attention, concentration, psychomotor speed, and visuo-spatial function are more affected in HCV patients than other domains.

This study had several limitations. Firstly, we faced inadequate funding resources. Secondly, sample population was relatively small to generalize the concept of associated cognitive impairment with higher hepatitis C virus infection. Administrative issues regarding interruption of case recruitment (frequent technical problems in MRI machine and software).

## Abbreviations

CHO: Choline
Cr: Creatine
Glu: Glutamate
HCV: Hepatitis C virus
mI: Myo-inosital
MMSE: Mini-mental state examination
MRS: Magnetic resonance spectroscopy
NAA: *N*-acetyl aspartate
PCR: Polymerase chain reaction
WMS-R: Wechsler Memory Scale - Revised
